# Immunosuppressive treatment in diffuse cutaneous systemic sclerosis is associated with an improved composite response index (CRISS)

**DOI:** 10.1186/s13075-020-02220-0

**Published:** 2020-06-05

**Authors:** Boyang Zheng, Marie Hudson, Mianbo Wang, Murray Baron, for the Canadian Scleroderma Research Group, Janet E. Pope, Murray Baron, Marie Hudson, Geneviève Gyger, Maggie J. Larché, Nader A. Khalidi, Ariel Masetto, Evelyn Sutton, David Robinson, Tatiana S. Rodriguez-Reyna, Nancy Maltez, Doug Smith, Carter Thorne, Alena Ikic, Paul R. Fortin, Marvin J. Fritzler

**Affiliations:** 1 Division of Rheumatology, Jewish General Hospital, McGill University, Montreal, QC Canada; 2 Jewish General Hospital, Lady Davis Institute for Medical Research, Montreal, QC Canada

**Keywords:** Systemic sclerosis, Scleroderma, Immunosuppression, Outcomes, CRISS

## Abstract

**Background:**

Outcomes of therapeutic studies in diffuse cutaneous systemic sclerosis (dcSSc) have mainly been measured for specific organs, particularly the skin and lungs. A new composite response index in dcSSc (CRISS) has been developed for clinical trials. The goal of this study was to determine whether, in an observational dcSSc cohort, immunosuppression was associated with global disease improvement measured with the CRISS.

**Methods:**

We conducted a retrospective cohort study in a multi-centered SSc registry comparing 47 patients newly exposed to immunosuppression for ≥ 1 year to 254 unexposed patients. Inverse probability of treatment weighting (IPTW) was performed to create comparable exposed and unexposed groups by balancing for age, sex, disease duration, modified Rodnan skin score (mRSS), forced vital capacity, patient and physician global assessments, and Health Assessment Questionnaire score. A CRISS score ≥ 0.6 at 1 year was defined as improvement.

**Results:**

Exposed patients had shorter disease duration (5.5 versus 11.7 years, *p* < 0.01), more interstitial lung disease (67.4% versus 40.3%, *p* < 0.01), and worse physician global severity scores (4.2 versus 2.5 points, *p* < 0.01) compared to unexposed patients. Improvement in CRISS scores was more common in exposed patients after IPTW (odds ratio 1.85, 95% confidence interval 1.11, 3.09). Of the individual CRISS variables, only mean patient global assessment scores were significantly better among exposed than unexposed patients (− 0.4 versus 0 points, *p* = 0.03) while other variables including mRSS were similar.

**Conclusion:**

Using a composite response measure, immunosuppression was associated with better outcomes at 1 year in a dcSSc cohort. These results provide real-world data that align with clinical trials to support our current use of immunosuppression.

## Background

Systemic sclerosis (SSc) is an autoimmune fibrosing disorder that affects the skin and internal organs. Diffuse cutaneous SSc (dcSSc) is characterized by skin thickening extending proximally to the elbows and knees and/or the trunk. It is associated with increased organ involvement, decreased patient function, and worse prognosis [1–5].

The mainstay of treatment in dcSSc is immunosuppression, including methotrexate (MTX), cyclophosphamide (CYC), azathioprine (AZA), and mycophenolate (MPA). Immunosuppression is posited to play a role in reducing early disease activity with the goal of preventing progression and irreversible damage [6]. Current guidelines suggest that immunosuppression should be considered in dcSSc patients [7], especially for the treatment of active lung, skin, or musculoskeletal manifestations [7, 8]. Clinical trials and other studies have shown benefits of immunosuppression on improving or at least stabilizing interstitial lung disease (ILD) with CYC [9] and MPA [10]. There is also some evidence for AZA as maintenance therapy in ILD [11]. MTX, which failed to meet clinical endpoints in one trial, was shown to benefit skin disease when the data was re-analyzed using Bayesian methods [12, 13].

While immunosuppressive therapies have been studied with regard to outcomes in specific organs, the benefit in overall disease has not been well evaluated because of the lack of adequate global measures. The American College of Rheumatology (ACR) approved a provisional composite response index in dcSSc (CRISS) [14] with the goal of improving outcome assessment in clinical trials by better capturing the multifaceted aspects of dcSSc. This measure includes disease areas that are susceptible to improve, including skin involvement based on the modified Rodnan skin score (mRSS), lung involvement based on the forced vital capacity (FVC), patient and physician global assessments of disease, and function based on the Health Assessment Questionnaire Disability Index (HAQ).

The CRISS was developed with a very specific goal, to assess global disease changes in dcSSc patients of < 5 years disease duration included in clinical trials. Although the CRISS was derived from observational dcSSc cohort data, whether it can measure response in this setting remains unknown. However, the main drawback of observational studies is the inherent confounding between treated and untreated groups. In recent years, advanced statistical methods such as propensity weighting, which can produce quasi-randomization, have been developed to overcome this limitation [15]. These methods have been used to ascertain treatment effects for otherwise difficult to answer questions in other SSc and rheumatology cohorts [16, 17].

The goal of this study was to determine whether immunosuppression was associated with global disease improvement in dcSSc in a real-world setting. We hypothesized that, in an observational dcSSc cohort, immunosuppression would be associated with improvement in overall disease measured with the CRISS after applying propensity weighting.

## Methods

### Study population

The Canadian Scleroderma Research Group (CSRG) registry recruits and follows patients from 15 centers in Canada and Mexico. These centers see local and regional referrals. All patients must have a diagnosis of SSc (confirmed by an experienced rheumatologist), be ≥ 18 years of age, provide informed consent, and be fluent in English, French, or Spanish. Over 98% of the cohort meets the 2013 ACR/EULAR classification criteria for SSc [18]. Patients with dcSSc defined by skin thickening proximal to the elbows or knees and/or trunk at any time enrolled between January 2005 and July 2017 were included in this study. Ethics committee approval for this study was obtained at the Jewish General Hospital, Montreal, Canada (approval number 2019-1597) and at all participating CSRG study sites.

### Exposure

Medication exposure was recorded yearly by study physicians and coded as current, past, or never. Patients exposed to immunosuppression (MTX, CYC, AZA, and MPA) at or prior to their initial entry visit into the CSRG (T0) were excluded. The *index visit* (T1) was defined as the first CSRG visit when exposure to immunosuppression was recorded. Patients were defined as exposed if they were also exposed at the subsequent annual visit (T2) following the index visit. One-year outcome using the CRISS was calculated comparing outcomes at T2 compared to T1. This way, we ensured that exposed patients had received between a minimum of 1 continuous year of treatment and up to a maximum of 2 years. Unexposed patients were those who had never been exposed to immunosuppression at or before CSRG entry and had at least two consecutive follow-up visits for which no treatment was recorded. Unexposed patients were matched to exposed patients based on the time since recruitment into the registry.

### Outcomes

The CRISS was developed to assess the likelihood of improvement after 1 year of observation [14]. It consists of two steps. Step 1 identifies patients with significant worsening or new end-organ damage. These patients are automatically defined as not improved and assigned a CRISS score of 0. The criteria for significant worsening or end-organ damage are as follows: new onset scleroderma renal crisis (SRC), new left heart failure with leftventricular ejection fraction ≤ 45% on transthoracic echocardiogram requiring treatment, new pulmonary arterial hypertension (confirmed on right heart catheterization) requiring treatment, ≥ 15% decline in FVC%, new interstitial lung disease (ILD) and FVC% below 80% predicted. Step 2 of the CRISS calculates an estimated improvement after 1 year using the CRISS equation. This takes into account the changes in: mRSS, percent predicted forced vital capacity (FVC%), patient and physician global assessment of disease severity, and HAQ-DI. A final CRISS score after both steps of ≥ 0.6 is considered as improved disease.

For individual CRISS variables, a categorical improvement after 1 year was defined by a favorable change in the absolute difference between measures at T1 and T2 as follows: mRSS change by ≥ 5 points [19], FVC % predicted by ≥ 5% [20], HAQ by ≥ 0.14 points [19], and patient and physician global assessments by ≥ 20% (≥ 2 points) based on previously used cutoffs [21].

### Definition of variables

Disease duration was defined from the onset of the first non-Raynaud’s phenomenon symptom to the index visit (T1). Smoking status was classified as either never smoker or past and/or current smoker. Skin involvement was assessed using the modified Rodnan skin score (mRSS), which ranges from 0 (no involvement) to 3 (severe thickening) in 17 areas (score range 0–51). FVC% was extracted from pulmonary function tests. The presence of ILD was determined using a published clinical decision rule [22]. Using this rule, ILD was considered present if a high-resolution computed tomography (HRCT) scan of the lung was interpreted by an experienced radiologist as showing ILD or, in the case where no HRCT was available, if either a chest X-ray was reported as showing either increased interstitial markings (not thought to be due to congestive heart failure) or fibrosis, and/or if a study physician reported the presence of typical “velcro-like crackles” on physical exam. Function was assessed using the HAQ-DI questionnaire which is scored from 0 (no disability) to 3 (severe disability). Patient and physician global assessment scores were rated 0–10 (no disease to very severe disease) on numeric rating scales. For patient assessment scores, patients were asked “in the past week, how was your overall health?”. The physician global severity question asked “How would you rate the patient’s overall health for the past week?”. Other covariates recorded at the index visit included physician reports of inflammatory myositis, arthritis, digital ulcers, prior scleroderma renal crisis, and the gastrointestinal-14 (GI-14) score, a summative score of 14 patient-reported symptoms [23]. The GI-14 correlates well with the UCLA Scleroderma Clinical Trial Consortium GI Tract Instrument [23]. Pulmonary hypertension was defined as an estimated systolic pulmonary artery pressure (sPAP) ≥ 45 mmHg measured using the Doppler flow measurement of the tricuspid regurgitant jet on cardiac echocardiography (used as a non-invasive screening tool for pulmonary hypertension) [24]. Antinuclear antibody was detected by immunofluorescence, and other autoantibodies were detected by line immunoassay (Euroimmun, Lübeck, Germany).

### Statistical analysis

Descriptive statistics were used to summarize baseline demographic and clinical characteristics of the exposed and unexposed patients. Continuous variables are presented as mean ± standard deviation (SD), and categorical variables are presented as counts and percentages. Student’s *t* test and Wilcoxon-Mann-Whitney *U* test were used to compare continuous variables. Chi-square test and Fisher exact test were used for categorical variables.

Due to the inherent differences between exposed and unexposed patients in an observational study, inverse probability of treatment weighting (IPTW) was used to balance the study groups [25]. Propensity scores were computed using conditional logistic regression by including age, sex, disease duration, and CRISS variables. The weights were calculated as the inverse of the propensity score and applied to each stratum of exposure and covariate. To evaluate residual differences in baseline covariates between the two groups, we calculated standardized differences for each variable. A standardized difference ≤ 0.1 represents meaningful balance [26]. The effect of the exposure on CRISS scores was assessed using weighted linear regression models which generated odds ratios (OR) with 95% confidence intervals (CI) adjusted for age, sex, and disease duration.

Missing data were imputed multiply and longitudinally 50 times at each observation using R version 3.2.0 for Windows (http://r-project.org). All other statistical analyses were performed with SAS v.9.4 (SAS Institute, USA).

## Results

**Fig. 1:**
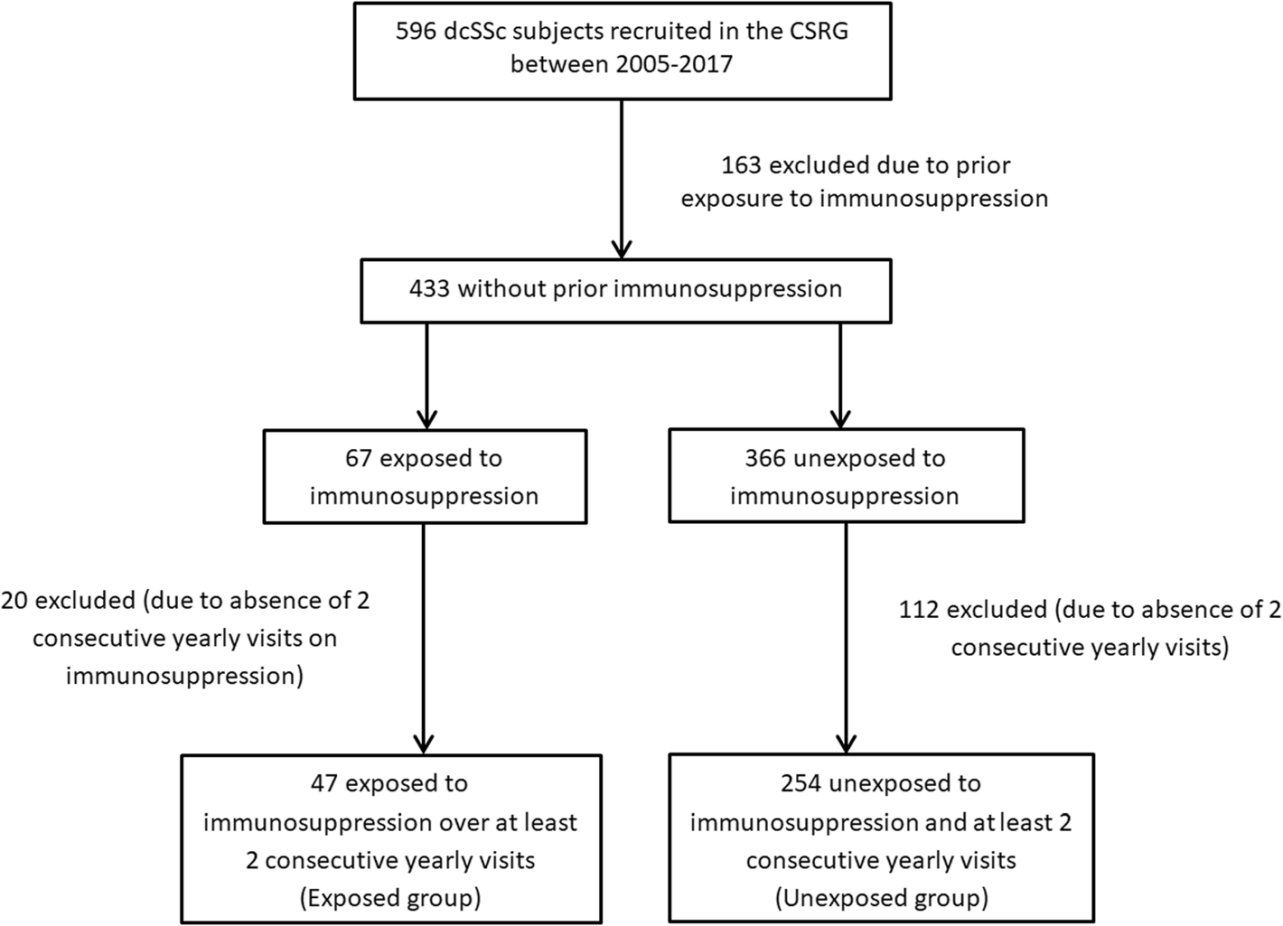
Study inclusion flow chart

There were 433 dcSSc patients without prior exposure to immunosuppression recruited in the CSRG cohort. Of these, 47 were exposed and 254 were unexposed patients for at least 2 consecutive follow-up visits (Fig. 1). None of the patients excluded were due to known deaths. Of the exposed group, 20 (42.6%), 15 (31.9%), 11 (23.4%), and 1 (2.1%) patient(s) were treated with MTX, MPA, CYC, and AZA, respectively. Exposed patients were younger (50.1 ± 10.4 versus 55.1 ± 12.3 years, *p* = 0.01), had shorter disease duration (5.5 ± 7.4 versus 11.7 ± 9.3 years, *p* < 0.01), had a higher prevalence of ILD (67.4% versus 40.3%, *p* < 0.01), and had worse physician global assessments of disease severity (4.2 ± 2.3 versus 2.5 ± 1.9 out of 10 points, *p* < 0.01) compared to unexposed patients (Table 1). Fewer exposed patients had anti-centromere antibodies (12.5% versus 27.6%, *p* = 0.04) or a history of inflammatory arthritis (27.7% versus 40.6%, *p* = 0.01).

**Table 1:** Comparison of patient characteristics at the index visit between the exposed and unexposed groups

	Exposed (*n* = 47)	Unexposed (*n* = 254)	*p*
**Patient characteristics**			
Median date of cohort entry, mm/yyyy	12/2008	02/2007	
Age, mean ± SD	50.1 ± 10.4	55.1 ± 12.3	0.01
Female, *n* (%)	37 (81.5%)	207 (78.7%)	0.66
Ethnicity, *n* (%)			0.39
Caucasian	36 (76.6%)	204 (81.6%)	
Aboriginal	1 (2.1%)	14 (5.6%)	
Black	1 (2.1%)	3 (1.2%)	
Asian	1 (2.1%)	5 (2.0%)	
Latin America	2 (4.3%)	4 (1.6%)	
Other	6 (12.8%)	20 (8.0%)	
Past and/or current smoker, *n* (%)	27 (62.8%)	148 (60.2%)	0.50
Disease duration (years), mean ± SD	5.5 ± 7.4	11.7 ± 9.3	< 0.01
Interstitial lung disease, *n* (%)	31 (67.4%)	102 (40.3%)	< 0.01
Pulmonary hypertension, *n* (%)^^^	4 (8.7%)	17 (7.5%)	0.76
Inflammatory myositis, *n* (%)	7 (14.9%)	36 (14.2%)	0.89
Inflammatory arthritis, *n* (%)	13 (27.7%)	103 (40.6%)	0.01
Digital ulcers, *n* (%)	29 (61.7%)	164 (64.6%)	0.71
Prior SRC, *n* (%)	5 (10.6%)	14 (5.5%)	0.32
GI-14 score, mean ± SD	2.6 ± 2.5	2.6 ± 2.5	0.93
Auto antibodies, *n* (%)*			
Anti-centromere	5 (12.5%)	64 (27.6%)	0.04
Anti-topoisomerase I	11 (27.5%)	40 (17.2%)	0.13
Anti-RNA polymerase III	11 (27.5%)	46 (19.8%)	0.27
Antinuclear antibody (titer ≥ 1/160)	38 (88.4%)	219 (92.4%)	0.37
**CRISS variables**			
mRSS, mean ± SD	15.0 ± 9.4	13.1 ± 9.4	0.15
FVC%, mean ± SD	88.5 ± 18.7	91.4 ± 17.6	0.36
HAQ, mean ± SD	0.8 ± 0.7	0.7 ± 0.7	0.65
PTGA disease severity, mean ± SD	3.5 ± 2.6	3.2 ± 2.2	0.58
PGA disease severity, mean ± SD	4.2 ± 2.3	2.5 ± 1.9	< 0.01

*SRC* scleroderma renal crisis, *GI-14 score* Canadian Scleroderma Research Group 14 point score for gastrointestinal involvement, *CRISS* composite response index in diffuse systemic sclerosis, *mRSS* modified Rodnan skin score, *FVC%* percent of predicted forced vital capacity, *HAQ* Health Assessment Questionnaire, *PTGA* patient global assessment, *PGA* physician global assessment

^^^Only 46 exposed and 227 unexposed patients had known pulmonary hypertension status

*Only 40 exposed and 232 unexposed patients had documented auto antibodies respectively

At 1 year, more patients in the exposed group had overall improvement, defined as a CRISS score ≥ 0.6, compared to the unexposed group (23.4% versus 11.8%, *p* = 0.03) (Table 2). This is in spite of the fact that more exposed patients had a CRISS = 0 because of significant organ worsening or end-organ damage than unexposed patients (10.6% versus 3.1%, *p* = 0.02). The only component of the CRISS that was significantly more improved in the exposed group was the mean patient global assessment score (− 0.4 ± 1.8 versus 0 ± 1.6, *p* = 0.03). The mean changes in other individual CRISS variables (mRSS, FVC%, HAQ-DI, and physician global assessment of disease severity) were small and not different between exposed and unexposed patients. Among subjects with a calculable CRISS (step 2) score, i.e., those who were not automatically assigned a CRISS = 0, the number of subjects with a categorical improvement in each disease measure was examined. The only CRISS variable that was different between exposed and unexposed groups was again patient global assessment scores where 42.9% of the exposed group had improved patient global compared to 26.4% of the unexposed group, *p* = 0.03 (Table 2). There were no deaths observed after 1 year.

**Table 2:** Comparison of the changes in individual CRISS variables and final CRISS outcome at 1 year between the exposed and unexposed groups

	Exposed, *n* = 47	Unexposed, *n* = 254	*p*
**Change in CRISS variables (mean ± SD)**			
mRSS	− 1.1 ± 6.4	− 0.3 ± 6.3	0.28
FVC %predicted	− 1.3 ± 11.1	− 0.4 ± 7.9	0.66
HAQ	− 0.1 ± 0.5	0 ± 0.3	0.56
PTGA disease severity	− 0.4 ± 1.8	0 ± 1.6	0.03
PGA disease severity	0.1 ± 2.1	− 0.1 ± 2.0	0.79
**CRISS outcome*n*(%)**			
Automatically not improved CRISS (step 1)*	5 (10.6%)	8 (3.1%)	0.02
Improved CRISS at 1 year	11 (23.4%)	30 (11.8%)	0.03
**Improvement among subjects with calculated CRISS (step 2)*n*(%)**^**#**^	*n* = 42	*n* = 246	
Improved mRSS ≥ 5	13 (31.0%)	50 (20.3%)	0.12
Improved FVC %predicted ≥ 5	11 (26.2%)	56 (22.8%)	0.63
Improved HAQ ≥ 0.14	13 (31.0%)	53 (21.5%)	0.18
Improved PTGA disease severity ≥ 2	18 (42.9%)	65 (26.4%)	0.03
Improved PGA disease severity ≥ 2	11 (26.2%)	85 (34.6%)	0.28

*mRSS* modified Rodnan skin score, *FVC%* percent of predicted forced vital capacity, *HAQ* Health Assessment Questionnaire, *PTGA* patient global assessment, *PGA* physician global assessment, *CRISS* composite response index in diffuse systemic sclerosis

*Of those considered not improved, these are patients with significant new or worsening organ involvement who were assigned an automatic CRISS score of 0 without the need for formulaic calculation of CRISS score

^#^Cutoffs represent the absolute difference in measures after 1 year towards an improved score

### Primary outcome in statistically weighted cohort

Statistical balance between exposed and unexposed groups was achieved with IPTW with standardized differences < 0.1 for all potentially confounding variables in the weighted populations (Table 3). In multivariate analyses, we found that prior to weighting, treatment already tended towards a higher likelihood of improvement with an OR (95% CI) of 2.00 (0.82, 4.92), *p* = 0.13 (Table 4). After weighting, exposed patients were significantly more likely to have improved disease measured with the CRISS than unexposed patients (OR (95% CI) 1.85 (1.11, 3.09), *p* = 0.02), adjusting for age, sex, and disease duration.

**Table 3:** Balancing effect of the propensity scores between exposed and unexposed groups

	Standardized difference* between groups before IPTW	Standardized difference* between groups after IPTW
Age	0.44	0.04
Female sex	0.07	0.02
Disease duration	0.73	0.08
mRSS	0.20	0.06
FVC %predicted	0.15	0.05
HAQ	0.07	0.02
PTGA disease severity	0.13	0.05
PGA disease severity	0.80	0.02

*IPTW* inverse probability of treatment weighting, *mRSS* modified Rodnan skin score, *FVC%* percent of predicted forced vital capacity, *HAQ* Health Assessment Questionnaire, *PTGA* patient global assessment, *PGA* physician global assessment

*Standardized difference ≤ 0.1 represents meaningful balance

**Table 4:** Multivariate analyses of the likelihood of improved CRISS with treatment before and after IPTW

	Before IPTW		After IPTW	
	OR (95% CI)	*p* values	OR (95% CI)	*p* values
Immunosuppression use	2.00 (0.82, 4.92)	0.13	1.85 (1.11, 3.09)	0.02
Female	1.07 (0.41, 2.79)	0.90	1.50 (0.73, 3.05)	0.26
Age	1.00 (0.97, 1.04)	0.86	1.03 (1.00, 1.05)	0.04
Disease duration	1.01 (0.96, 1.05)	0.80	1.00 (0.97, 1.02)	0.81

*IPTW* inverse probability of treatment weighting, *OR (95% CI)* odds ratio with 95% confidence interval

### Post hoc analysis

A post hoc analysis was performed to examine the impact of patient global assessment scores on the CRISS, since there may be a placebo effect. Exposed patients may have been influenced to report more global improvement due to the unblinded nature of the study and was the only variable that was different between CRISS improvers and non-improvers. We tested the robustness of our findings using a “worst-case” scenario. All subjects with an improved patient global at 1 year were assigned a change in patient global = 0 (i.e., no change), while those with worsening patient global scores were unchanged. In this analysis, the association between treatment and improved CRISS after IPTW persisted, with an OR (95% CI) of 1.86 (1.07, 3.24), *p* = 0.03 (Table 5).

**Table 5:** Multivariate analyses of the likelihood of improved CRISS with treatment before and after IPTW after recoding patient global assessment scores

	Before IPTW		After IPTW	
	OR (95% CI)	*p* values	OR (95% CI)	*p* values
Immunosuppression use	2.08 (0.78, 5.57)	0.14	1.86 (1.07, 3.24)	0.03
Female	0.89 (0.32, 2.47)	0.83	1.25 (0.58, 2.63)	0.56
Age	1.00 (0.97, 1.04)	0.84	1.03 (1.00, 1.06)	0.04
Disease duration	1.02 (0.97, 1.06)	0.47	1.01 (0.98, 1.03)	0.57

*IPTW* inverse probability of treatment weighting, *OR (95% CI)* odds ratio with 95% confidence interval

A second post hoc analysis was performed to examine whether patients with improved patient global scores might have more improvement in the other CRISS features. We found that among the exposed patients, those who had improved patient global scores by at least 2 points had significantly better mean change in FVC% (2.2 ± 7.6% improvement versus a − 2.8 ± 6.4% decline, *p* = 0.04). However, no correlation was found between improved patient global and other mean disease scores when the overall group of both exposed and unexposed patients was assessed. These are shown in supplementary Table 1.

## Discussion

This is the first study to investigate global outcomes in an observational cohort of dcSSc patients using the CRISS. As expected, patients exposed to immunosuppression had worse disease compared to unexposed patients, reflecting confounding by indication. However, after weighting observations by the probability of treatment, we were able to balance the groups and thus correct for baseline differences in the exposed and unexposed groups. We found that immunosuppressive treatment was a significant predictor of improvement at 1 year. This was despite the fact that the exposed group was sicker and had more patients with substantially worsened disease after 1 year. Indeed, even if there was residual confounding, our findings of improved CRISS in the exposed group may be conservative estimates of the truth.

It is important to note that, except for the patient global assessment scores, none of the other individual CRISS variables showed statistically significant differences in favor of exposure. The main concern with regard to the validity of this finding remains the possibility of a placebo effect. Since treatment was not blinded, exposure to immunosuppression may have biased patients to report improvement. We therefore performed a post hoc analysis that would address this bias and found that this was not the case. The fact that patient global assessment scores did not significantly impact our findings is in keeping with the original CRISS cohort where patient global assessment was the least predictive of improved CRISS [14]. The difference in patient global scores might otherwise be due to improvement in domains that we did not measure such as pain or fatigue or that the patient’s appreciation of their own disease is more sensitive than other measures. In a second post hoc analysis, we found that FVC% change was more favorable among exposed patients who also reported an improved patient global score than those who did not have an improved patient global. Although this finding is limited by the small number of patients in this subgroup, it suggests that bias from non-blinded exposure to immunosuppression was not the only reason that exposed patients reported global disease improvement.

Our results lend weight to the idea that the mean change in any individual disease measure such as mRSS and FVC may not be representative of the heterogeneous disease changes in a patient. There were significantly more CRISS improvers in spite of little to no difference in the mean change of individual CRISS variables between the exposure groups. It may be that the combination of changes within an individual is more significant than the pooled mean of a specific outcome measure. This supports the concept that a combined response index may be more valuable than organ-specific outcomes [12, 14] and this deserves further examination. Such measures are frequently used in other rheumatologic conditions such as rheumatoid arthritis and lupus for which core outcome measures are composite indices [27, 28].

With regard to immunosuppression, previous studies have shown the effects to be modest at best. One example is the effect of methotrexate on skin involvement which failed to meet trial endpoints until the data was re-analyzed using Bayesian methods [12, 13]. In other cases such as lung disease, immunosuppression achieved only FVC stability [9–11]. Because exposed patients had more severe baseline disease and more significant disease worsening (automatic assigned CRISS score = 0), the lack of worsening of individual CRISS measures among the exposed may be significant in and of itself. However, it is impossible to infer causality or to separate the effects of treatment from the natural course of disease stability. Supporting the idea that a global disease measure may be more appropriate than individual measures to assess response to immunosuppression is a recent observational study by the ESOS group which found no difference in skin changes between exposed and unexposed dcSSc patients, although survival was better among the exposed [29]. Low-dose cyclophosphamide has also shown some benefit when evaluated using another global measure, the Medsger disease severity score [30]. Further support is seen in aggressive treatment with high-dose immunosuppression [31] and in the context of stem cell transplant [32] which has shown improvement in various disease endpoints including global disease measures such as the global rank composite score (GRCS) [33] and the European Scleroderma Research Group Activity Index (EScSG-AI) [34].

### Limitations

Assessing the validity of the CRISS was not our goal. The reported sensitivity and specificity of this score for improvement was 98.2% and 93.1%, respectively [14]. In addition, it has been shown to have good face and content validity and good sensitivity to change [35, 36]. Another limitation is that our sample population encompassed a wider spectrum of patients than those for which the CRISS was developed. In particular, the average disease duration was long in both groups, i.e., 5.5 ± 7.4 and 11.7 ± 9.3 years among the exposed and unexposed groups, respectively, whereas the mean disease duration of the CRISS cohort was 2.36 ± 1.5 years [14]. Although the CRISS was created using observational patient cohorts with < 5 years disease duration, the items and weights were developed by asking experts to rate a patient’s improvement given a list of disease variable changes after 1 year. There is no compelling reason to suspect that these conclusions should differ in patients with longer disease duration but showing the same pattern of change.

We were also limited by the low number of exposed patients in the study (*n* = 47) because patients with prior immunosuppression were excluded (see Fig. 1). This selection also contributed to the longer disease durations, suggesting that our patients may have had a less progressive disease course. Furthermore, certain aspects of dcSSc such as skin involvement tend to stabilize or regress after 5 years. This may account for the lower baseline mRSS scores and explain why only minimal differences were seen in objective disease measures after 1 year between the exposure groups. Despite adjusting for disease duration, exposed patients had earlier disease and thus a higher likelihood of spontaneous regression that is impossible to differentiate from treatment effects. Nevertheless, the use of propensity weighting adjusted for major imbalances between the 2 groups and allowed the assessment of treatment in an observational cohort such as ours. Medication exposure in the registry was assessed at yearly intervals without details regarding dose and start/stop dates. While we do not know the exact duration of treatment, we selected patients who were exposed for at least 2 consecutive yearly visits in order to reduce potential misclassification bias. Finally, while IPTW balancing was statistically valid, we recognize that there may be residual confounding inherent with the observational study design.

## Conclusion

After applying the CRISS in a real-world setting beyond the intended clinical trial population, our findings reinforce the hypothesis that immunosuppression may improve overall disease. Further, our findings that organ-specific outcomes did not differ between CRISS improvers and non-improvers may reflect the concept that a composite score within an individual is a more sensitive measure than the aggregate mean of organ-specific measures across individuals.

## Supplementary information

**Additional file 1: Supplementary Table 1.** Comparison of the changes in individual CRISS variables at 1 year between those with and without improved patient globalassessment scores.

## Data Availability

The data that support the findings of this study are available from the CSRG but restrictions apply to the availability of these data, which were used under license for the current study, and so are not publicly available. Data are however available from the authors upon reasonable request and with permission of the CSRG.

## Abbreviations

ACR: American College of Rheumatology
AZA: Azathioprine
CRISS: Composite response index in dcSSc
CSRG: Canadian Scleroderma Research Group
CYC: Cyclophosphamide
dcSSc: Diffuse cutaneous systemic sclerosis
FVC: Forced vital capacity
GI-14: Gastrointestinal-14 score
HAQ: Health Assessment Questionnaire Disability Index
HRCT: High-resolution computed tomography
ILD: Interstitial lung disease
IPTW: Inverse probability of treatment weighting
MPA: Mycophenolate
mRSS: Modified Rodnan skin score
MTX: Methotrexate
sPAP: Systolic pulmonary artery pressure
SSc: Systemic sclerosis
