# Supplementary material for: Immunosuppressive treatment in diffuse cutaneous systemic sclerosis is associated with an improved composite response index (CRISS)
Source: Arthritis Res Ther. 2020 Jun 5;22:132. doi: 10.1186/s13075-020-02220-0 (PMC7275378; doi:10.1186/s13075-020-02220-0)
Supplement: Supplementary file 1 — Additional file 1: Supplementary Table 1. Comparison of the changes in individual CRISS variables at 1 year between those with and without improved patient globalassessment scores. [file 13075_2020_2220_MOESM1_ESM.docx]

Supplementary document

Supplementary Table 1: Comparison of the changes in individual CRISS variables at 1 year between those with and without improved patient global assessment scores

| **Exposed subjects with a calculated CRISS (step 2) score** | | | |
| --- | --- | --- | --- |
| Change in CRISS variables (mean ± SD) | Patients with improved patient global score ≥ 2* (n=18) | Patients without improved patient global score ≥ 2^#^ (n=24) | P values |
| mRSS | -2.0 ± 5.0 | -1.2 ± 5.7 | 0.75 |
| FVC %predicted | 2.2 ± 7.6 | -2.8 ± 6.4 | 0.04 |
| PGA disease severity | 0.3 ± 2.3 | 0.2 ± 1.8 | 0.99 |
| HAQ | -0.1 ± 0.4 | -0.1 ± 0.5 | 0.90 |
| **All subjects with a calculated CRISS (step 2) score** | | | |
|  | Patients with improved patient global score by ≥ 2 points (n=85) | Patients without improved patient global score by ≥ 2 points (n=203) |  |
| mRSS | -1.4 ± 6.3 | -0.1 ± 5.9 | 0.17 |
| FVC %predicted | 0.7 ± 6.8 | -0.2 ± 7.6 | 0.46 |
| PGA disease severity | -0.2 ± 2.2 | 0.1 ± 1.9 | 0.42 |
| HAQ | -0.1 ± 0.4 | 0.0 ± 0.4 | 0.17 |
| mRSS = modified Rodnan skin score, FVC%= percent of predicted forced vital capacity, HAQ = Health Assessment Questionnaire, PGA = physician global assessment, CRISS = composite response index in diffuse systemic sclerosis  *This includes all patients with an improved global assessment score of ≥2 points at 1 year  ^#^ This includes all patients with a stable, worse, or less than 2 point improvement in patient global assessment score at 1 year | | | |
